# Environmental and lifestyle factors in multiple sclerosis progression

**DOI:** 10.3389/fimmu.2026.1971890

**Published:** 2026-10-26

**Authors:** Anna Karin Hedström

**Affiliations:** Department of Clinical Neuroscience, Karolinska Institutet, Stockholm, Sweden

**Keywords:** disease progression, environmental exposures, host genetics, immune regulation, lifestyle factors, metabolic dysfunction, multiple sclerosis, neuroinflammation

## Abstract

Multiple sclerosis (MS) is characterized by marked heterogeneity in inflammatory activity, disability accumulation, and long-term clinical outcomes. Although disease-modifying therapies have substantially improved control of inflammatory disease activity, disability progression remains incompletely explained by treatment exposure and conventional clinical predictors. Environmental and lifestyle factors are increasingly recognized as potential modifiers of disease course after MS onset, but they are often studied in isolation. In this review, we examine evidence linking persistent viral infections, inhalational exposures, sun exposure and vitamin D, obesity, diet and the gut microbiota, physical activity and sleep characteristics to MS disease activity and progression. Across these exposures, several shared pathways emerge, including chronic innate immune activation, persistent adaptive immune stimulation and impaired immune regulation, metabolic and mitochondrial dysfunction, oxidative stress, and altered capacity for tissue repair and neuroimmune resilience. We also discuss evidence that environmental effects may depend on host genetic background, particularly human leukocyte antigen (HLA) class I and class II variation. Although causal evidence remains incomplete and many studies are limited by reverse causation, residual confounding, and exposure misclassification, integrating environmental, genetic, immune, and metabolic data may help explain heterogeneity in MS outcomes and identify opportunities for more personalized prevention and intervention after disease onset.

## Introduction

Multiple sclerosis (MS) is a chronic immune-mediated disease of the central nervous system characterized by marked heterogeneity in clinical course, disease activity, and long-term outcomes (1). Some individuals experience minimal disability accumulation over decades, whereas others develop substantial neurological impairment despite access to modern disease-modifying therapies (DMTs) (2). Understanding the factors that contribute to this variability has become a major research priority, particularly because disability progression may occur independently of overt inflammatory disease activity (3–5).

The mechanisms underlying disability are likely multifactorial. Chronic compartmentalized inflammation, microglial activation, mitochondrial dysfunction, oxidative stress, and impaired tissue repair have all been implicated in disease worsening (6, 7). Environmental and lifestyle factors may influence these processes and thereby contribute to variation in disease activity, recovery, and long-term disability accumulation (8).

Gene-environment interactions have been extensively studied in relation to MS susceptibility (9), but considerably less attention has been directed toward their potential role after disease onset. Genetic variation may influence responses to environmental and lifestyle exposures and help explain why individuals exposed to similar risk factors can experience markedly different clinical trajectories.

In this review, we examine evidence that environmental and lifestyle factors influence disease activity and progression after MS onset. We focus on exposures with clinical or radiological relevance after diagnosis and discuss the immune and neurobiological pathways through which these factors may act. We also consider how genetic background may modify the effects of environmental exposures and propose an integrated framework for understanding variability in long-term MS outcomes.

## Conceptual framework for environmental and lifestyle modifiers of MS progression

Environmental and lifestyle exposures associated with MS outcomes have been linked to alterations in immune regulation, cellular metabolism, oxidative stress, neuroinflammation, and mechanisms of tissue repair (6, 7). These observations suggest that apparently distinct exposures may influence MS disease course through partly overlapping biological pathways. Rather than considering each factor in isolation, environmental exposures can therefore be viewed in relation to the disease-relevant processes they affect.

We propose a framework in which environmental and lifestyle exposures influence disease activity and progression through interconnected immune and neurobiological pathways (**Figure 1**). The relative importance of these pathways is likely to differ between exposures and across disease stages. Genetic variation may further amplify or attenuate biological responses to environmental factors, thereby modifying their effects on progression-relevant pathways.

**Figure 1:**
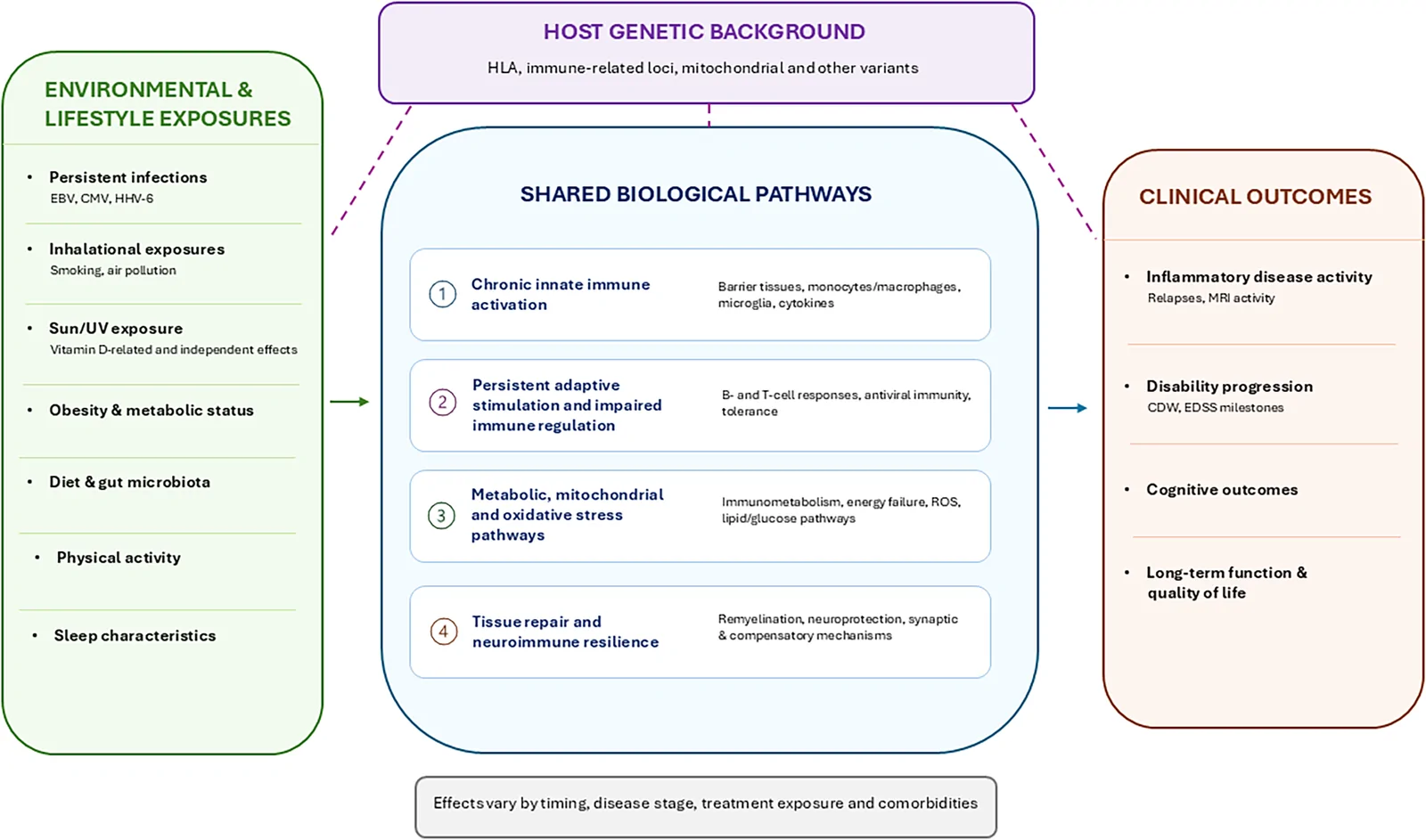
Shared biological processes linking environmental and lifestyle factors to MS outcomes. Environmental and lifestyle factors associated with MS outcomes may influence disease activity and progression through partially overlapping biological processes, including altered immune regulation, metabolic dysfunction, oxidative stress, neuroinflammation, and mechanisms of tissue repair and neuroimmune resilience. Genetic variation may modify biological responses to these exposures, thereby contributing to heterogeneity in clinical trajectories and long-term outcomes. This framework is intended as a conceptual model and does not imply that all factors act through identical mechanisms or contribute equally to disease progression.

This framework provides a basis for integrating environmental, genetic, and immunological perspectives on MS progression. The following sections examine individual environmental and lifestyle exposures within this broader biological context.

## Persistent viral infections and host immune responses

### Epstein-Barr virus

Epstein-Barr virus (EBV) is a ubiquitous gammaherpesvirus that establishes lifelong infection and persists predominantly within the memory B-cell compartment, where it remains under continuous immune surveillance (10, 11). Periodic viral reactivation and latency-associated effects on B-cell survival, activation, and antigen presentation may provide opportunities for recurrent antigenic stimulation and sustained interaction with host immune pathways (11, 12). The balance between viral persistence and immune control varies between individuals and is influenced by genetic, environmental, and immunological factors (13).

### Host immune responses to Epstein-Barr virus

Primary EBV infection is efficiently controlled in most individuals through coordinated innate and adaptive immune responses (14, 15). When primary infection is delayed beyond childhood, symptomatic infection is more likely and may present as infectious mononucleosis (IM) (15, 16). IM is characterized by a pronounced cellular immune response directed against EBV-infected B cells and is widely considered an immunopathological consequence of delayed primary infection rather than a direct effect of viral replication alone (16–18). During acute IM, marked activation and expansion of EBV-specific CD8+ T cells are accompanied by activation of cytotoxic CD4+ T cells, natural killer cells, and extensive cytokine production (14, 15), reflecting the intensity of the antiviral immune response. The development of IM illustrates substantial inter-individual variation in host responses to EBV and is influenced by age at infection, genetic variation, and the efficiency of antiviral immune control (13, 18, 19).

### Epstein-Barr virus and MS progression

After disease onset, EBV may remain relevant through effects on B-cell survival and activation, antigen presentation, and virus-driven immune stimulation (20). In addition, molecular mimicry between EBV antigens and CNS proteins, including GlialCAM, CRYAB, and ANO2, has been proposed as a possible link between antiviral immunity and MS pathobiology (20–22).

Compared with healthy individuals, patients with MS consistently exhibit enhanced humoral and cellular immune responses to EBV (23, 24). Higher antibody levels, particularly against EBNA1, have been associated with greater MRI activity in some cohorts (25), while more recent studies have linked EBV-specific antibody responses to choroid plexus inflammation and brain volume (26, 27). However, relatively few studies have examined disability progression directly, and results across cohorts have been conflicting (27–29). These discrepancies likely reflect differences in study design, disease stage, follow-up duration, outcome measures, and the specific EBV antigens examined.

Although elevated EBNA1 antibody responses are strongly associated with MS susceptibility (30–32), their interpretation in established disease is less straightforward. Recent findings suggest that the association between EBNA1 antibody levels and disability progression may vary according to host immunogenetic background rather than representing a universal marker of progression (33).

Evidence relating EBV-specific responses and markers of viral reactivation to long-term clinical outcomes remains limited. Although altered peripheral and intrathecal EBV-specific immune responses and evidence of viral reactivation have been reported in subsets of patients with MS (24, 34), a prospective study of patients with clinically isolated syndrome found no association between serial EBV IgG levels and subsequent clinical or MRI outcomes over five years (35). Similarly, reductions in anti-EBV IgG during ocrelizumab treatment were not associated with clinical or MRI outcomes (36). Recent neuropathological findings further indicate that B-cell-rich perivascular inflammation can occur irrespectively of detectable local EBV (37). Overall, current evidence does not establish that persistent EBV infection directly drives MS progression, but it supports continued investigation into whether persistent virus-host interactions contribute to heterogeneity in disease activity, compartmentalized inflammation, and long-term disability accumulation.

### Other persistent viral infections

Immune responses to other persistent herpesviruses, particularly cytomegalovirus (CMV) and human herpesvirus 6 (HHV-6), have been investigated in relation to MS course. In a prospective cohort of patients with newly diagnosed MS, stronger CMV-specific immune responses were associated with a lower risk of long-term disability accumulation and inflammatory disease outcomes (38). These findings are supported by subsequent data linking stronger CMV immune responses to lower serum neurofilament light chain levels and reduced risk of disability accumulation (39). In contrast, higher or increasing HHV-6 antibody levels have been associated with increased relapse risk or disability progression (29, 40, 41). However, not all studies have confirmed these findings, and evidence of HHV-6 or EBV reactivation has not consistently been associated with subsequent clinical course (42). CMV infection has been associated with changes in the diversity and phenotype of EBV-specific CD8+ T cells, while HHV-6 can activate the EBV lytic cycle *in vitro (*43, 44). Whether these interactions influence MS progression remains unknown.

Together, these findings suggest that host immune responses to persistent viral infections may contribute to disease heterogeneity in MS, although the available evidence remains limited.

## Inhalational exposures and the lung-brain immune axis

The respiratory tract is an important site of immune regulation. Continuous exposure of the airway mucosa to inhaled environmental agents can influence both innate and adaptive immune responses, making the lung a plausible site where environmental exposures shape systemic autoimmunity. Cigarette smoking provides the strongest evidence for this concept, although air pollution and other inhalational exposures have also attracted increasing attention.

### Tobacco smoke exposure

Cigarette smoking is among the environmental exposures with the strongest evidence for an adverse effect on MS disease course. Early longitudinal studies linked smoking to a less favorable disease course, including increased risk of conversion to secondary progressive MS and accelerated radiological worsening, with faster increases in T2 lesion volume and greater loss of brain parenchymal fraction (45, 46). Subsequent cohort studies demonstrated that smokers also experience more rapid disability accumulation and poorer long-term clinical outcomes (45–49). Smoking has also been associated with adverse radiological and cognitive outcomes, including greater gray matter atrophy and poorer cognitive performance (49–53). However, studies conducted specifically in primary progressive MS have generally not shown a clear association between smoking and disability worsening (54, 55). Whether this reflects biological differences between disease phenotypes or differences in study design and patient populations remains uncertain. Overall, smoking has been associated with both inflammatory disease activity and neuroaxonal injury, including gray matter atrophy, although the available studies do not clearly distinguish its associations with relapse-associated worsening from those with progression independent of relapse activity.

Several observational studies have shown that patients who stop smoking subsequently experience slower disability accumulation than those who continue smoking, although their prognosis often remains less favorable than that of never smokers (49, 56–58). While residual confounding cannot be excluded, the consistency of these findings supports smoking cessation as a highly plausible secondary preventive intervention in MS. Passive smoking has also been associated with greater disability at diagnosis and more rapid progression in a large nationwide cohort (49), extending the relevance of tobacco smoke beyond active smoking alone.

The adverse effects of smoking appear to remain clinically relevant despite the widespread use of modern DMTs. Across several treatments, including interferon-β, fingolimod, dimethyl fumarate, natalizumab, and ocrelizumab, smokers have generally experienced greater disease activity or less favorable clinical outcomes than non-smokers (59–62). In addition, smoking has been associated with an increased risk of anti-drug antibodies, including neutralizing antibodies against interferon-β and anti-natalizumab antibodies (63–65).

### Ambient air pollution and other inhalational exposures

Compared with cigarette smoking, evidence for ambient air pollution and other inhalational exposures is more limited. Several studies have linked ambient air pollution to increased inflammatory disease activity in MS, including clinical relapses, relapse-related hospitalizations, and MRI inflammatory activity (66–70). Most studies have focused on particulate matter, although ozone has also been independently associated with relapse risk after adjustment for co-pollutant exposures (71). Evidence for long-term disability progression is based on a small number of heterogeneous observational studies, with substantial variability in exposure assessment and disability outcome definitions, precluding firm conclusions regarding the role of air pollution in long-term disability accumulation (72).

Evidence regarding other inhalational exposures is even more limited. Occupational exposure to organic solvents, industrial dust, and other airborne exposures has been implicated in MS susceptibility (73, 74), but potential effects on subsequent disability progression remain largely unexplored.

### Mechanisms linking inhalational exposures to MS progression

The respiratory mucosa contains a dense network of epithelial cells, alveolar macrophages, dendritic cells, and tissue-resident lymphocytes that continuously survey the external environment while maintaining immune homeostasis (75). Chronic exposure to cigarette smoke or air pollutants may disrupt this balance, resulting in persistent innate immune activation, increased production of pro-inflammatory cytokines, and oxidative stress (76–78). Bronchoalveolar lavage studies have identified increased preformed CD40L expression in airway CD4+ T cells from nonsmoking patients with MS, while smoking was associated with increased T-cell proliferation in the deep airways (79).

Experimental studies have demonstrated that the lung serves as a site of immune programming before CNS infiltration. In experimental autoimmune encephalomyelitis, autoreactive CD4+ T cells transiently accumulate within the lung, where they undergo functional reprogramming before acquiring the capacity to enter the CNS (78). Recent work suggests that the lung microenvironment may also promote metabolic remodeling of CD4+ T cells, including increased fatty acid uptake and β-oxidation-dependent effector differentiation, thereby enhancing their encephalitogenic potential (80). These observations indicate that the pulmonary environment can actively shape the functional state of autoreactive lymphocytes.

Although these mechanisms were initially proposed to explain how environmental exposures may contribute to CNS autoimmunity, they may also be relevant after MS onset. Chronic pulmonary immune activation could contribute to systemic inflammatory signaling and thereby provide a link between inhalational exposures and inflammatory disease activity in MS. Whether these pathways contribute directly to long-term disability progression remains less certain.

Emerging evidence suggests that pulmonary immune activation may also be influenced by the lung microbiome. Experimental manipulation of pulmonary microbial communities can modify CNS immune reactivity and microglial activation, at least partly through type I interferon-dependent pathways (81). Together, these findings support the concept of a lung–brain immune axis, whereby signals originating in the respiratory tract may influence CNS inflammatory responses.

## Sun exposure and vitamin D

The relationship between sun exposure and MS progression has received less attention than its role in MS susceptibility, but the available evidence generally supports an association between lower sun exposure and less favorable disease outcomes. An early study among veterans with progressive MS reported that greater premorbid sun exposure was associated with slower disability progression (82). This finding was subsequently supported by an observational study showing associations between sunlight exposure, sun sensitivity, and disability progression, although reverse causality could not be excluded (83). More recently, analyses from a large Swedish nationwide cohort have shown that low sun exposure at or near the time of diagnosis is associated with increased risks of confirmed disability worsening and EDSS progression (84), with similar findings reported in an independent prospective cohort (85). An imaging study further reported an association between greater sun exposure and larger whole-brain and gray matter volumes independently of circulating vitamin D levels (86). Although the number of independent cohorts remains limited, the available findings consistently support an association between lower sun exposure and less favorable long-term MS outcomes.

Vitamin D has been extensively investigated as a potential determinant of MS disease course, but the evidence is more heterogeneous. Higher 25-hydroxyvitamin D concentrations have been associated with lower relapse rates and less MRI evidence of inflammatory activity, particularly early in the disease course (87–90). In contrast, associations with long-term disability progression, brain atrophy, and other neurodegenerative MRI outcomes have been less consistent (89–93). Overall, vitamin D is more consistently associated with inflammatory disease activity than with long-term disability progression.

Intervention studies have produced less consistent support than observational studies (94). Several randomized trials have evaluated vitamin D supplementation in MS, with most failing to demonstrate convincing benefits on disability progression or other long-term clinical outcomes, although modest effects on inflammatory disease activity have been reported in some studies (95–97). A recent trial in individuals with clinically isolated syndrome suggested a reduction in inflammatory disease activity with high-dose vitamin D (98), but whether this translates into improved long-term clinical outcomes remains uncertain. Clinical studies of narrowband UVB phototherapy remain limited and have generally been underpowered to evaluate clinical efficacy (99, 100).

Although closely related, sun exposure and vitamin D should be considered distinct exposures. Epidemiological studies suggest that the association between sun exposure and MS progression may be stronger and more consistent than that observed for circulating vitamin D concentrations, underscoring the importance of evaluating these factors separately.

### Mechanisms linking sun exposure and vitamin D to MS progression

UV exposure modulates innate and adaptive immune responses through both vitamin D-dependent and vitamin D-independent pathways (101). Vitamin D influences several immunological processes relevant to MS, including inhibition of pro-inflammatory T-cell responses, promotion of regulatory T-cell function, modulation of dendritic cell maturation, and reduced production of inflammatory cytokines (102, 103).

UV exposure also induces immunoregulatory mediators in the skin, promotes tolerogenic dendritic cells, expands regulatory immune-cell populations, and suppresses pro-inflammatory cytokine production (101). Experimental models further support UV-mediated suppression of neuroinflammation independently of vitamin D production and vitamin D receptor signaling (104, 105).

Narrowband UVB phototherapy has induced reproducible immunological changes in individuals with clinically isolated syndrome and early MS, including alterations in immune-cell function, inflammatory cytokine production, proteomic signatures, and circulating neurofilament light concentrations (100, 106, 107). A recent single-cell study extended these findings by demonstrating UVB-induced transcriptional and migratory changes in immune cells across the skin and peripheral blood, with preliminary evidence of changes in the CSF. The propagation of immune responses from the skin to the blood appeared attenuated in participants with RRMS compared with healthy controls (108). Although these studies were not designed or powered to establish effects on long-term clinical progression, the observed effects on immune-cell trafficking, cytokine production, and regulatory signaling provide evidence for immunomodulatory pathways extending beyond vitamin D synthesis.

## Obesity and metabolic dysfunction

The available evidence generally supports an association between obesity and less favorable long-term prognosis in MS. Prospective cohort studies have reported that obesity at or near the time of diagnosis is associated with more rapid disability accumulation and an increased risk of reaching disability milestones (109–112). Similar associations have been observed when body mass index (BMI) is analyzed as a continuous measure (111, 112), although not all studies have confirmed these findings (91, 113, 114). In addition, studies of disability accumulation have generally not distinguished relapse-associated worsening from progression independent of relapse activity, making it difficult to determine whether obesity is associated primarily with relapse-associated or relapse-independent disability worsening, or both. Adjustment for potential confounding varied between studies. Most longitudinal analyses accounted for age, sex, smoking, disease characteristics, and disease-modifying treatment, whereas physical activity was considered only in some studies and diet and the gut microbiome were not included (110–113). Residual confounding therefore remains possible, although these factors may, depending on their timing, act as confounders, mediators, or consequences of disability. Evidence for MRI and cognitive outcomes is less consistent. Several studies have reported increased inflammatory MRI activity, greater brain volume loss, and more rapid cognitive decline among individuals with higher BMI (111, 115, 116), whereas other cohorts have not confirmed these associations (113, 117–119).

A limited number of studies have suggested that obesity may be associated with reduced effectiveness of first-line injectable DMTs, including interferon-β and glatiramer acetate, although evidence is limited to relatively small adult cohorts and pediatric populations (120, 121).

Whether weight reduction after MS onset modifies disease progression remains uncertain. Observational data do not establish that weight loss after diagnosis improves disability progression, cognitive outcomes, or patient-reported outcomes (111), and a nationwide matched cohort study found no evidence that bariatric surgery improves long-term MS outcomes (122). However, longitudinal data suggest that excess adiposity in early adulthood is associated with subsequent disease course mainly when elevated BMI persists until MS diagnosis (112), raising the possibility that the timing and persistence of excess adiposity are relevant to long-term disease progression.

### Mechanisms linking obesity and metabolic dysfunction to MS progression

Adipose tissue functions as an active endocrine and immunological organ that produces cytokines, adipokines, and lipid mediators capable of influencing both innate and adaptive immune responses (123, 124). In obesity, adipose tissue inflammation promotes a chronic low-grade inflammatory state characterized by increased levels of pro-inflammatory cytokines and adipokines, including leptin, resistin, and visfatin, together with relative reductions in anti-inflammatory adipokines such as adiponectin (124). These changes may favor pro-inflammatory T-cell differentiation, impair regulatory T-cell function, and contribute to sustained immune activation relevant to MS (124). Recent translational work suggests that these effects may be sex dependent. Obesity was associated with a circulating protein signature enriched for Th1, IL-17, and MS-related pathways selectively among women, while diet-induced obesity worsened EAE in female but not male mice through type I interferon-dependent enhancement of Th1 responses (125). Whether these findings translate into sex differences in obesity-associated disability progression in people with MS remains uncertain.

Obesity also alters cellular metabolism, including insulin signaling, mitochondrial function, oxidative stress, and lipid metabolism, thereby influencing immune-cell activation, differentiation, and effector function (123, 126). Metabolic alterations may additionally induce epigenetic changes in immune-cells (127). In individuals with MS, obesity has been associated with ceramide-dependent DNA methylation changes in monocytes, higher monocyte counts, greater MRI lesion burden, and worse clinical disease course, raising the possibility that chronic metabolic dysfunction may shape immune responses through epigenetic reprogramming (115).

Experimental and clinical studies further suggest that obesity-related metabolic dysfunction may influence CNS processes relevant to MS, including compartmentalized inflammation, microglial activation, oxidative stress, blood-brain barrier dysfunction, and impaired myelin repair (128–131).

## Diet and gut microbiota

Cross-sectional and observational studies have associated greater adherence to healthy dietary patterns, including Mediterranean-style diets and diets rich in fruits, vegetables, whole grains, and unsaturated fats, with lower disability and reduced symptom burden (132–134). Evidence linking specific dietary patterns to long-term disability has been inconsistent across cohorts (135). A prospective study reported that higher diet quality was associated with less subsequent disability progression over 7.5 years (136), but the evidence remains sparse and vulnerable to reverse causation, residual confounding, and reliance on self-reported dietary intake.

Interest has also focused on the gut microbiota as a potential mediator of dietary effects. Altered gut microbial composition has frequently been reported in people with MS compared with healthy controls, but considerable heterogeneity exists between studies, and no reproducible disease-specific microbial signature has emerged (137–140). More recently, prospective and translational studies have shifted attention toward microbial function, linking short-chain fatty-acid-producing microbes, bile acid metabolism, and tryptophan-derived metabolites to subsequent disability progression, conversion to secondary progressive MS, cognition, and brain atrophy (141, 142). These findings suggest that microbial function may be more relevant than taxonomic composition alone.

Intervention studies of dietary and microbiota-targeted approaches, including probiotics, prebiotics, gut-derived metabolites, and fecal microbiota transplantation, have demonstrated immunological and metabolic effects in early studies (143), but convincing evidence that they modify long-term disability progression is lacking.

### Mechanisms linking diet and gut microbiota to MS progression

Diet shapes the composition and function of the gut microbiota, altering the production of bioactive metabolites, including short-chain fatty acids, bile acids, and tryptophan-derived metabolites (143). These metabolites can regulate innate and adaptive immune responses and influence CNS-relevant processes implicated in MS, including blood–brain barrier integrity, glial activation, oxidative stress, and mechanisms of myelin repair (143–145).

Specific metabolite pathways provide mechanistic examples of gut–brain immune signaling. Tryptophan-derived microbial metabolites can regulate microglial control of astrocyte pathogenic activity through aryl hydrocarbon receptor-dependent pathways, with potential consequences for CNS inflammation (144). Short-chain fatty acids may also be relevant to tissue repair, as butyrate has been shown experimentally to suppress demyelination, promote oligodendrocyte differentiation, and enhance remyelination (145).

Bile acids may influence glial activation, cytokine signaling, neuronal survival, and neuroinflammatory pathways through receptors expressed on immune and CNS cells (146), and altered bile acid metabolism has recently been associated with an increased risk of confirmed disability worsening in people with newly diagnosed MS (147). These findings provide plausible pathways linking dietary habits, gut microbial metabolism, and long-term MS outcomes, although direct evidence that dietary modification alters disability progression remains limited.

## Physical activity and neuroimmune resilience

Physical activity is widely recommended as part of comprehensive MS care because of its well-established benefits for physical function, fatigue, mood, and quality of life (148, 149). Whether physical activity influences long-term disability accumulation or neurodegenerative outcomes is less certain, and interpretation is complicated because declining physical activity may itself represent an early manifestation of disability progression (150, 151). Nevertheless, studies assessing physical activity before or near disease onset have reported slower subsequent disability progression among more physically active individuals (152, 153), reducing but not eliminating concerns about reverse causation.

Evidence for MRI and cognitive outcomes remains mixed. Observational studies and randomized exercise trials have reported favorable effects on selected MRI measures, including regional brain volumes and microstructural integrity (154–156), but findings are inconsistent and trials have generally not shown convincing effects on global brain atrophy, lesion load, or relapse-related outcomes (156–158). Cognitive outcomes have likewise been heterogeneous across trials and meta-analyses (159–163).

### Mechanisms linking physical activity to MS progression

Exercise may influence neurotrophic signaling, particularly through brain-derived neurotrophic factor (BDNF), which is involved in neuronal survival, synaptic plasticity, and myelin repair (164, 165). In people with MS, exercise interventions have been associated with increases in peripheral BDNF concentrations (166), although evidence linking these changes directly to neuroprotection or slower disability progression remains limited.

Exercise further induces metabolic adaptations that may support neuronal resilience, including improved mitochondrial function, enhanced oxidative phosphorylation, reduced oxidative stress, and systemic anti-inflammatory effects (167, 168). Recent experimental studies have also identified the exercise-induced myokine irisin as a potential mediator of neuroprotective effects (169). Exercise-related signals may additionally modulate stromal-immune interactions. Mechanical stress activates Piezo1-dependent inflammatory programs in skeletal-muscle stromal cells, while irisin increases IL-33 production by adipose stromal cells and preserves ST2+ regulatory T cells in obese male mice (170, 171). The relevance of these pathways to MS progression remains unknown.

## Sleep characteristics

Sleep disturbances are highly prevalent in people with MS and have often been studied in relation to fatigue, mood symptoms, symptom burden, and quality of life rather than as potential contributors to disease progression (172, 173). Emerging evidence suggests that sleep characteristics may also have prognostic relevance. An early longitudinal study reported that poor sleep quality was associated with a less favorable disability course (174). More recently, a population-based prospective study of incident MS found that poor sleep quality at diagnosis was associated with increased risks of confirmed disability worsening and earlier attainment of EDSS milestones, independent of baseline disability, treatment exposure, and other established prognostic factors (175). Short sleep duration (<7 hours/night) showed similar associations, whereas social jetlag, as a marker of circadian misalignment, was not consistently associated with progression (175).

Intervention studies have primarily examined symptom-related outcomes. Mindfulness- and exercise-based approaches have improved sleep in some studies, although effects vary across interventions and sleep metrics (176–178). Whether improving sleep can influence long-term disability progression remains unknown.

### Mechanisms linking sleep characteristics to MS progression

Sleep disruption can alter innate and adaptive immune responses and promote a pro-inflammatory state, including increased production of pro-inflammatory cytokines (179, 180).

Sleep also supports processes relevant to CNS resilience, including glymphatic clearance, synaptic homeostasis, and neuroendocrine regulation (181–183). Impaired sleep has been linked to oxidative stress, mitochondrial dysfunction, and dysregulation of the hypothalamic–pituitary–adrenal axis, mechanisms that may plausibly increase neuroaxonal vulnerability (181–184).

Circadian disruption may further influence immune-cell trafficking and inflammatory activity through alterations in circadian clock signaling, although its specific role in MS progression remains incompletely understood (185).

## Shared biological pathways linking environmental exposures to MS progression

The environmental and lifestyle factors discussed in this review differ substantially in their nature, timing, and degree of modifiability. Persistent viral infections, inhalational exposures, obesity, sun exposure, physical activity, diet, and sleep are often studied as separate risk or prognostic factors. However, their biological effects are unlikely to operate in isolation. Instead, these exposures appear to converge on a limited number of interconnected immune and neurobiological pathways relevant to MS progression (**Table 1**, **Figure 1**). These include chronic innate immune activation, persistent adaptive immune stimulation and impaired immune regulation, metabolic and mitochondrial dysfunction, and reduced capacity for tissue repair and neuroimmune resilience (186–190).

**Table 1:** Shared biological pathways and genetic context linking environmental and lifestyle factors to MS progression.

Shared pathway or modifying context	Core biological features	Illustrative environmental/lifestyle exposures and related biological states	Potential relevance to MS outcomes	Key uncertainties
Chronic innate immune activation	Low-grade inflammatory signaling involving monocytes, macrophages, dendritic cells, microglia, astrocytes, epithelial barrier tissues, cytokine production, and barrier-related immune activation	Smoking, air pollution, obesity, sleep disruption, diet-related gut microbial changes	May lower the threshold for inflammatory disease activity and contribute to compartmentalized CNS inflammation	Whether peripheral innate activation directly contributes to long-term disability progression remains difficult to separate from associations with relapse activity
Persistent adaptive immune stimulation and impaired immune regulation	Sustained B- and T-cell activation, persistent viral antigenic stimulation, altered antiviral surveillance, impaired regulatory T-cell function, altered effector-regulatory balance	EBV and other herpesviruses, obesity, diet-related metabolic changes, low UV exposure, vitamin D status	May influence inflammatory disease activity and longer-term progression by maintaining immune activation or weakening regulatory control	EBV-related markers may reflect immune control rather than causal viral activity; clinical relevance may depend on HLA background.
Metabolic, mitochondrial, and oxidative stress pathways	Immunometabolic reprogramming, altered lipid and glucose metabolism, mitochondrial dysfunction, impaired energy supply, and oxidative injury	Obesity, physical inactivity, diet, sleep disruption, smoking, air pollution	May promote inflammatory immune responses and reduce the capacity of neurons, oligodendrocytes, and axons to withstand chronic injury	Causal directionality is difficult to establish, and these pathways are highly interconnected with inflammation and repair mechanisms.
Tissue repair and neuroimmune resilience	Remyelination, neuroprotection, synaptic adaptation, glymphatic clearance, neurotrophic signaling, mitochondrial resilience, compensatory capacity	Physical activity, sleep, UV exposure, vitamin D status, dietary patterns and microbial metabolites; negatively influenced by smoking and obesity	May determine whether inflammatory or metabolic injury translates into irreversible disability accumulation	Direct human evidence that modifying these pathways slows disability progression remains limited
Genetically conditioned exposure responses	HLA-mediated antigen presentation, antiviral immune surveillance, type I interferon signaling, and broader immune-regulatory context	Particularly relevant for smoking, obesity, low UV exposure, and EBV-related antibody responses	May explain why the same exposure has different prognostic relevance across individuals	Requires replication and integration with immune, metabolic, genetic, and longitudinal biomarkers

CNS, central nervous system; EBV, Epstein-Barr virus; HLA, human leukocyte antigen; MS, multiple sclerosis; ROS, reactive oxygen species; UV, ultraviolet. Environmental and lifestyle factors are illustrative, and pathways are not mutually exclusive.

### Chronic innate immune activation

A first shared pathway is chronic activation of innate immune responses. Cigarette smoke, ambient air pollution, obesity, sleep disruption, and gut microbial dysbiosis can all promote low-grade inflammatory signaling involving monocytes, macrophages, dendritic cells, microglia, astrocytes, and epithelial barrier tissues (76, 77, 124, 126, 180). Inhaled toxicants may initiate immune activation at the respiratory mucosa, where epithelial cells and resident immune cells respond to particulate matter, oxidative stress, and tissue injury (75, 77). Obesity similarly induces chronic adipose tissue inflammation, with increased production of inflammatory cytokines and adipokines that shape systemic immune responses (126). Sleep disruption and circadian dysregulation may further amplify innate inflammatory signaling by altering cytokine production, immune-cell trafficking, and neuroendocrine regulation (179, 180, 183, 185). Although these exposures act through different entry points, they may converge on a common state of persistent innate immune activation that lowers the threshold for inflammatory disease activity and may contribute to compartmentalized CNS inflammation.

### Persistent adaptive immune stimulation and impaired immune regulation

A second shared pathway involves sustained adaptive immune activation and impaired immune regulation (7, 186). EBV provides the clearest example of a persistent antigenic stimulus capable of shaping long-term B-cell and T-cell responses (10, 15, 33). EBV persists within memory B cells, can undergo periodic reactivation, and may influence B-cell survival, antigen presentation, and antiviral immune surveillance throughout life (11, 12, 15). Other persistent herpesviruses, including CMV and HHV-6, may also influence disease heterogeneity, although the evidence remains more limited (29, 38, 39, 41). Obesity and diet-related metabolic changes may further promote pro-inflammatory T helper cell differentiation and impair regulatory T-cell function (125, 126, 191), while reduced UV exposure and low vitamin D status may weaken regulatory immune pathways (99, 103). Together, these exposures may shift the balance between effector and regulatory adaptive immune responses, thereby influencing inflammatory activity and longer-term progression.

### Metabolic and mitochondrial dysfunction

Metabolic dysfunction represents another central point of convergence. Obesity, physical inactivity, unhealthy dietary patterns, sleep disruption, and chronic inflammation can all influence mitochondrial function, oxidative stress, lipid metabolism, insulin signaling, and cellular energy balance (123, 126, 167, 184). These processes are relevant because both immune-cell activation and CNS tissue maintenance are metabolically demanding. Activated immune cells undergo metabolic reprogramming to support proliferation and effector function (192), while neurons, oligodendrocytes, and axons depend on intact mitochondrial function and energy supply for long-term survival (187, 193). Metabolic stress may therefore act at both systemic and CNS-compartmentalized levels, promoting inflammatory immune responses while simultaneously reducing the capacity of neural tissue to withstand injury.

Several exposures also converge on oxidative stress and mitochondrial injury. Cigarette smoke and air pollution directly introduce oxidants and pro-oxidant stimuli, while obesity and sleep disruption promote oxidative stress through inflammatory and metabolic pathways (76, 77, 126, 184). Mitochondrial dysfunction has been implicated in progressive MS pathology and may contribute to axonal vulnerability, particularly in the setting of chronic inflammation (7, 187). Conversely, physical activity may improve mitochondrial function, enhance oxidative phosphorylation, and reduce systemic inflammatory tone (167–169). These opposing patterns suggest that lifestyle factors may influence progression partly by shifting the balance between mitochondrial injury and metabolic resilience.

### Tissue repair and neuroimmune resilience

A further shared mechanism concerns tissue repair and neuroimmune resilience. Progression in MS is not determined solely by the extent of inflammatory injury, but also by the capacity for recovery, remyelination, synaptic adaptation, and neuroaxonal survival (188, 189, 194). Several environmental factors may influence this balance. Physical activity may promote neurotrophic signaling, mitochondrial resilience, and direct neuroprotective pathways (165, 166, 169). Adequate sleep may support glymphatic clearance, synaptic homeostasis, and neuroendocrine regulation (181–183). UV exposure and vitamin D may promote regulatory immune responses that reduce inflammatory tissue injury (101, 103). Diet and gut microbial metabolites may influence myelin repair, glial activation, and blood–brain barrier integrity (143–145). In contrast, chronic inflammation, obesity, smoking, poor sleep, and metabolic dysfunction may impair repair processes and increase neuroaxonal vulnerability (7, 188). This framework shifts attention from progression as a purely degenerative process to progression as the result of an imbalance between injury and resilience.

These pathways are highly interconnected. Chronic innate immune activation can promote adaptive immune responses; persistent adaptive stimulation can maintain innate activation; metabolic dysfunction can amplify inflammatory signaling; and inflammation can impair mitochondrial function and tissue repair (7, 186, 192). For example, obesity may simultaneously promote adipose inflammation, alter adipokine signaling, reprogram immune-cell metabolism, and impair CNS repair mechanisms (124–126, 131). Inhaled environmental exposures, including cigarette smoke and ambient air pollution, may act through pulmonary immune activation, oxidative stress, epithelial barrier responses, and systemic inflammatory effects (76, 77). Sleep disruption may influence cytokine production, circadian immune regulation, glymphatic clearance, and neuroendocrine stress pathways (180, 181, 183, 185). Diet may act through systemic metabolism, gut microbial metabolites, immune regulation, and glial function (124, 144, 145). These examples illustrate why environmental exposures should not be interpreted as isolated predictors, but as components of broader biological networks. The observation that smoking and obesity interact additively in relation to disability progression and cognitive decline (195) further supports the concept that lifestyle-related exposures may share or amplify common pathogenic pathways.

The timing of exposure is likely to be important. Some exposures may exert their strongest effects before disease onset or early in the disease course, when inflammatory activity is high and long-term trajectories may still be modifiable (194). Others may remain relevant throughout established disease by influencing ongoing inflammation, treatment response, comorbidity burden, or repair capacity (57, 59, 168, 196). For example, obesity or low sun exposure near diagnosis may influence the early biological environment in which long-term disability trajectories are established (109–111, 197). Smoking may remain relevant after diagnosis through persistent pulmonary and systemic immune activation, as well as possible effects on treatment response (57, 60, 77). Physical activity, sleep, and diet may fluctuate over time and may both influence and be influenced by evolving disability, creating particular challenges for causal interpretation (136, 153, 175). These temporal considerations highlight the need for repeated exposure assessment and longitudinal biomarker data.

### Genetic context and exposure-specific vulnerability

An important implication of this framework is that convergence on shared biological pathways does not imply uniform effects across individuals. Genetic variation may influence how environmental exposures are translated into immune activation, metabolic stress, tissue injury, or repair responses. This is particularly relevant in MS, where genetic effects on susceptibility are well established (9), but genetic determinants of progression have been more difficult to identify (190, 198, 199). The limited marginal effects of individual genetic variants on disability progression do not exclude a role for genetics in shaping responses to environmental exposures.

Emerging evidence suggests that HLA class I and class II variation may modify the prognostic relevance of several environmental and lifestyle factors after MS onset. HLA-A*02:01 is of particular interest because it is associated with reduced MS susceptibility and has established roles in antigen presentation to CD8+ T cells, antiviral immune surveillance, and broader immune regulatory pathways (32, 198, 200, 201). Recent studies suggest that the adverse associations of smoking, obesity, and low sun exposure with disability progression may be more pronounced among individuals lacking HLA-A*02:01 (109, 197, 202). Similarly, the prognostic value of EBV-related antibody responses appears to depend on combined HLA-A*02:01 and HLA-DRB1*15:01 status (33), indicating that markers of persistent virus–host interaction may not have uniform meaning across immunogenetic backgrounds.

These findings suggest that environmental exposures may act within genetically conditioned immune contexts. The absence of HLA-A*02:01 may mark a state in which inflammatory or metabolic stressors are more readily translated into sustained immune activation, impaired immune regulation, or reduced resilience to tissue injury. This does not necessarily imply that HLA-A*02:01 itself is the causal mediator. The HLA region is characterized by extensive linkage disequilibrium, and observed associations may reflect broader differences in antigen presentation, antiviral responses, type I interferon signaling, or other immune regulatory pathways (198, 201, 203). Nevertheless, the repeated observation that several distinct exposures show stronger adverse associations among HLA-A*02:01-negative individuals (33, 109, 197, 202) supports the concept of genetically modified environmental vulnerability in MS progression.

This perspective also has methodological implications. Studies of environmental modifiers of MS progression may underestimate or obscure associations if genetic heterogeneity is not considered. Apparent inconsistencies across cohorts may partly reflect differences in genetic background, exposure distribution, disease stage, treatment context, or outcome definitions. These considerations are important when comparing studies and interpreting null or heterogeneous findings.

This integrated model has several broader implications. First, environmental and lifestyle factors may influence MS progression through shared mechanisms even when individual epidemiological associations are modest. Second, interventions targeting one exposure may have broader biological effects if they influence common pathways such as inflammation, metabolism, sleep regulation, or immune resilience. Third, the same exposure may not have the same prognostic relevance in all individuals, because biological responses may depend on genetic background, disease stage, treatment exposure, and comorbid conditions. Finally, the distinction between inflammatory disease activity and progression should not be interpreted too rigidly. Several environmental exposures may influence both inflammatory activity and mechanisms of tissue injury or repair, thereby bridging processes that are often studied separately (7, 190, 194).

### Future directions

Progress in this field will require moving beyond single-exposure analyses toward integrated approaches that capture the complexity of environmental and lifestyle influences across the MS disease course. The exposome encompasses environmental and lifestyle exposures across the life course, together with the biological responses they induce. Exposome studies combine questionnaires and registry data with geospatial and occupational information, wearable or portable sensors, and biomarkers of exposure and biological response (204, 205). Combining these data may help characterize cumulative and time-varying exposure patterns, including combinations of smoking, air pollution, diet, physical activity, sleep, ultraviolet exposure, and infections. Relating these complex exposure profiles to MS progression will require repeated exposure assessment, harmonized outcome definitions, and longitudinal follow-up beginning close to disease onset.

Integration with multi-omics and cellular technologies will be important for linking epidemiological associations to disease-relevant mechanisms. Genomics, epigenomics, transcriptomics, proteomics, metabolomics, microbiome profiling, and immune phenotyping may help identify the biological pathways through which environmental exposures influence inflammatory activity, metabolic stress, tissue injury, and repair (194, 205). Single-cell and spatial approaches could further clarify how systemic exposures are reflected in specific immune-cell states, glial responses, and CNS-compartmentalized processes (206). These technologies can generate mechanistic hypotheses. Experimental systems, including animal models, can then test how specific exposures alter inflammatory activity, tissue injury, and repair under controlled conditions.

A central priority is to establish whether modifying environmental and lifestyle factors can alter long-term outcomes in people with MS. This will require pragmatic trials and target-trial emulation using high-quality observational data, with mechanistic biomarkers incorporated alongside clinical outcomes (228). For most exposures discussed, the available evidence is insufficient to establish consistent sex differences in associations with disability progression. Sex-specific estimates and formal interaction analyses are needed. Future work should also evaluate whether exposure and intervention effects differ according to genetic background, sex, disease stage, treatment exposure, and comorbidity burden. Ultimately, integrating environmental, genetic, immune, metabolic, and clinical data may support more precise prognostic models and help identify individuals most likely to benefit from targeted lifestyle or environmental interventions.

Taken together, the available evidence supports a model in which environmental exposures influence MS progression through interacting immune, metabolic, and neurobiological pathways. Causal evidence remains incomplete for several exposures, and reverse causation, residual confounding, and measurement limitations remain important concerns. However, the convergence of epidemiological, experimental, and translational findings suggests that disease progression is shaped not only by intrinsic disease biology and treatment exposure, but also by modifiable factors that affect inflammatory activity, metabolic stress, immune regulation, and neuroimmune resilience. Understanding these shared pathways, and the genetic contexts in which they operate, may help explain heterogeneity in long-term outcomes and identify opportunities for personalized prevention and intervention after MS onset.
